# Quantification of fractal dimension and Shannon’s entropy in histological diagnosis of prostate cancer

**DOI:** 10.1186/1472-6890-13-6

**Published:** 2013-02-18

**Authors:** Pedro Francisco Ferraz de Arruda, Márcio Gatti, Fernando Nestor Facio Junior, José Germano Ferraz de Arruda, Roberto Douglas Moreira, Luiz Otávio Murta, Laísa Ferraz de Arruda, Moacir Fernandes de Godoy

**Affiliations:** 1 Faculdade de Medicina de São José do Rio Preto (FAMERP), Av. Brig. Faria Lima 5416, 15090-000, São José do Rio Preto, SP, Brazil; 2 Disciplina de Cardiologia da FAMERP, São José do Rio Preto, Brazil; 3 Faculdade de Filosofia, Ciências e Letras de Ribeirão Preto da Universidade de São Paulo (FFCLRP-USP), Ribeirão Preto, Brazil; 4 União das Faculdades dos Grandes Lagos (Unilago), São José do Rio Preto, Brazil; 5 Departamento de Cardiologia e Cirurgia Cardiovascular da FAMERP, São José do Rio Preto, Brazil; 6 Núcleo Transdisciplinar para Estudo do Caos e da Complexidade (NUTECC-CNPq), São José do Rio Preto, Brazil

**Keywords:** Prostate cancer, Diagnosis, Fractal dimension, Shannon’s entropy

## Abstract

**Background:**

Prostate cancer is a serious public health problem that affects quality of life and has a significant mortality rate. The aim of the present study was to quantify the fractal dimension and Shannon’s entropy in the histological diagnosis of prostate cancer.

**Methods:**

Thirty-four patients with prostate cancer aged 50 to 75 years having been submitted to radical prostatectomy participated in the study. Histological slides of normal (N), hyperplastic (H) and tumor (T) areas of the prostate were digitally photographed with three different magnifications (40x, 100x and 400x) and analyzed. The fractal dimension (FD), Shannon’s entropy (SE) and number of cell nuclei (NCN) in these areas were compared.

**Results:**

FD analysis demonstrated the following significant differences between groups: T vs. N and H vs. N groups (p < 0.05) at a magnification of 40x; T vs. N (p < 0.01) at 100x and H vs. N (p < 0.01) at 400x. SE analysis revealed the following significant differences groups: T vs. H and T vs. N (p < 0.05) at 100x; and T vs. H and T vs. N (p < 0.001) at 400x. NCN analysis demonstrated the following significant differences between groups: T vs. H and T vs. N (p < 0.05) at 40x; T vs. H and T vs. N (p < 0.0001) at 100x; and T vs. H and T vs. N (p < 0.01) at 400x.

**Conclusions:**

The quantification of the FD and SE, together with the number of cell nuclei, has potential clinical applications in the histological diagnosis of prostate cancer.

## Background

Prostate cancer is a serious public health problem that affects quality of life, has a significant mortality rate and is ranked as the fifth most frequent form of cancer worldwide. [1] This disease affects elderly individuals more, with peak incidence and mortality at around 70 years of age, accounting for 10 to 30% of clinical tumors found in men and 60% of all male deaths due to cancer. [2] Autopsies performed on men report frequency of 10% of this type of cancer at 50 years of age and 40% at 70 years of age. Males between 70 and 80 years of age have a 15% chance of exhibiting clinically detected prostate cancer and a 3% risk of death by this form of neoplasm [3].

The diagnosis of prostate cancer is based on clinical (rectal touch), laboratory (prostate-specific antigen [PSA]) and radiological (ultrasound and computed tomography) exams, which may indicate the need for a transrectal biopsy. Despite doubts regarding diagnostic value, the early detection of this type of carcinoma is one of the most important clinical aspects in urology. Nonetheless, there is a doubt of placing too much value on the diagnosis and treatment, considering the possibility of the non-occurrence of the clinical development of the disease [4].

While annual PSA and rectal touch exams nearly always detect cancer prior to the development of metastasis, the benefits of population screening campaigns for the detection of initial tumors remain debatable. The PSA exam is criticized due to its lack of specificity and the detection of tumors with low biological aggressiveness, which may not cause symptoms or progress over the course of several years. There are also questions regarding which PSA values lead to the indication of biopsy when other parameters are normal. Once cancer is detected, the volume, exact location and extension of the tumor and its histological grade are difficult to determine with precision [5]. Thus, there is a need to seek methods that assist in the diagnosis of this disease.

Fractal dimension analysis has recently been used in a number of fields of medicine, such as cardiology, neurology, ophthalmology and radiology, due mainly to technological advances in computer science [6]. The fractal dimension is a useful parameter for the characterization of complex, irregular structures, the analysis of which, when examined mathematically, denotes figures with self-similarity (figures that resemble themselves when examined on different size scales) [7]. Using fractal analysis on biopsies of dog mammary glands, Simeonov & Simeonova [8] found a significant difference between the tumor area and benign tissue. In urology, the analysis of the fractal dimension has been used in the study of prostate tumor tissue [7] as well as vascularization in tumors of the prostate and around the parenchyma of the prostate gland [1].

Thus, a study on the application of fractal dimension analysis is warranted in the differential diagnosis of prostate cancer from other prostate conditions. To our knowledge, few studies are found in the international literature. Moreover, considering the facts that treatment decisions in such cases are hindered by the imprecise determination of the clinical stage, biopsy results underestimate the extent of the cancer and diagnostic imaging exams exhibit a low degree of specificity, patients with this type of cancer may benefit from the analysis of the fractal dimension in the diagnosis, which can allow avoiding the overtreatment of patients who will not go on to develop clinical complications in the future.

The aim of the present investigation was to study the quantification of the fractal dimension, Shannon’s entropy and the number of cell nuclei in the histological diagnosis of prostate cancer.

## Methods

Thirty-four patients of different races aged 50 to 75 years (mean: 64.4 ± 5.9 years) with PSA values between 4.7 to 26.1 ng/mL, having been submitted to radical prostatectomy for prostate cancer participated in the study. The patients were treated by the Urology Service of the university hospital of the *Faculdade de Medicina de São José do Rio Preto* (FAMERP, Brazil) between 2007 and 2008. All patients had the clinical stage determined, had tumors in the prostate with Gleason´ s scale varying between 6 and 9, staging between pT2a to pT3a and were residents of the region surrounding São José do Rio Preto. The study received approval from the FAMERP ethics committee (Protocol 1153/2010); the ethics committee waived the need for us to obtain informed consent from patients.

Histological slides of fragments taken from normal, hyperplastic and tumor tissues of the same prostate (same patient) were analyzed. This material was obtained from the Slide Bank of the Pathology Laboratory of the Department of Pathology and Forensics, FAMERP. The material was fixed in a 10% formalin solution and embedded in paraffin. Serial cuts measuring 5 to 6 mm in thickness were obtained and the fragments were then stained with hematoxylin-eosin.

The microscopic images were captured digitally using a Samsung SCC-131 digital camera coupled to an Olympus BX41 trinocular microscope, with a 10x plan achromatic objective with an Olympus U-TV1X-2 adaptor at a resolution of 800 × 600 (total magnification: 400x). The images were stored in jpeg, which allows storing high-resolution images in relatively small files. Each slide was photographed with three different magnifications (40x, 100x and 400x) and saved in jpeg format for the subsequent fractal dimension analysis.

**Figure 1:**
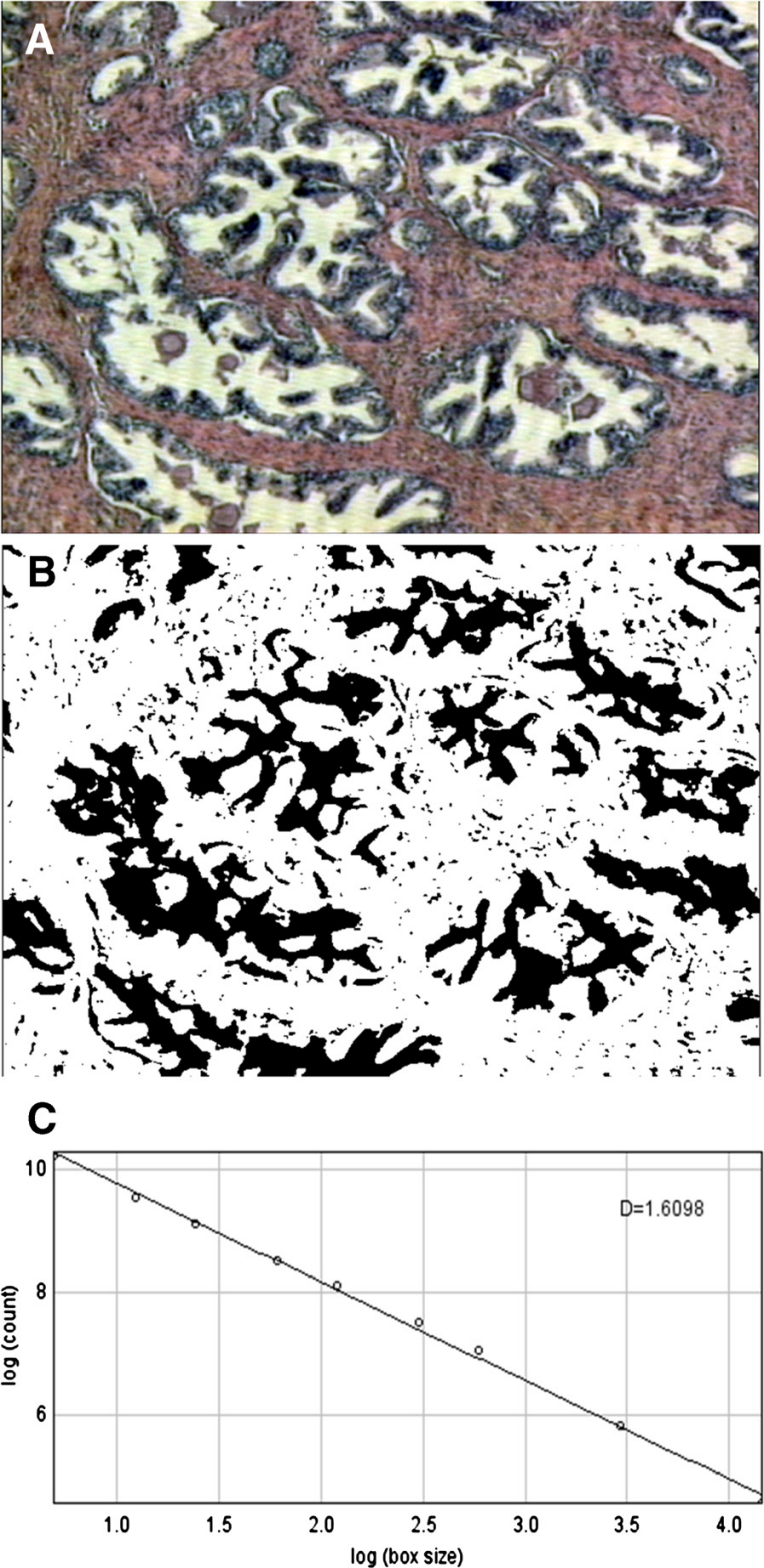
Procedure for construct the outlined images: (A) example of original histological section of hematoxylin-eosin hyperplastic tissues of the same prostate (same patient) (magnification x40), (B) binary image after segmentation into features of interest and background, and (C) outlined image used for box-counting FD calculation.

The fractal dimension was estimated by Box-counting method, using the software ImageJ (National Institute of Health, Bethesda, USA), widely used in the literature and available free on the Internet (http://rsbweb.nih.gov/ij/ ). This software considers the Box-counting in two dimensions, allowing the quantification of the distribution of pixels harvest area, not considering, therefore, the texture image. The influence of this is that two images with the same distribution of pixels, and another in a binarized gray levels, possess the same fractal dimension. The binarisation process of RGB images was performed using a fix threshold for all images. For this, the FD will be calculated with the ImageJ always between 0 and 2, not distinguishing different textures (Figure 1). The program received further implementation of the physicist and engineer LOMJ with plugins that facilitated the collection of results as a whole, including the possibility of simultaneous study of the Shannon Entropy and Counting of Cell Nuclei.

The fractal dimension, entropy and number of cell nuclei obtained from tissue unaffected by tumors were statistically compared with findings in hyperplastic and tumor tissues using the Kruskal-Wallis test. The statistical analysis was performed using the StatsDirect program, version 1.9.15 (StatsDirect Limited).

## Results

**Figure 2:**
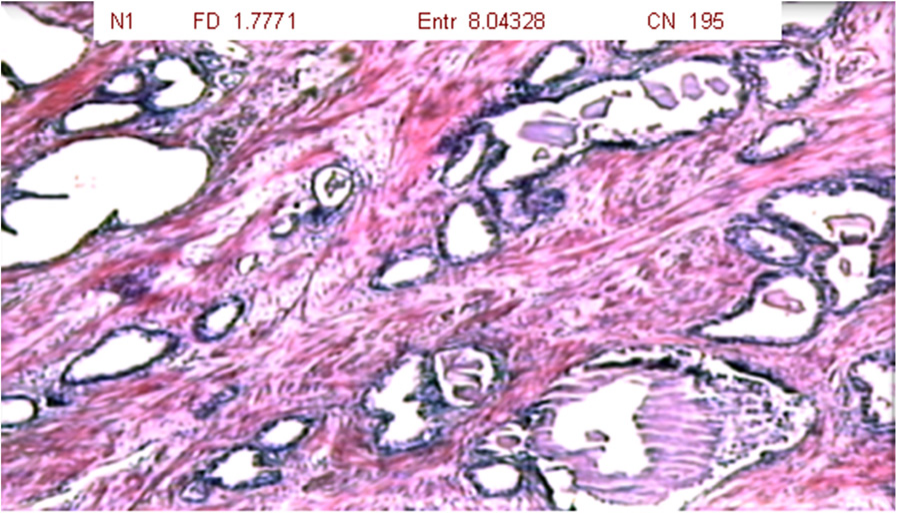
Photomicrograph of normal prostate tissue showing basal cells with no infiltrative aspects or clusters of acini (magnification: 40x; HE staining).

**Figure 3:**
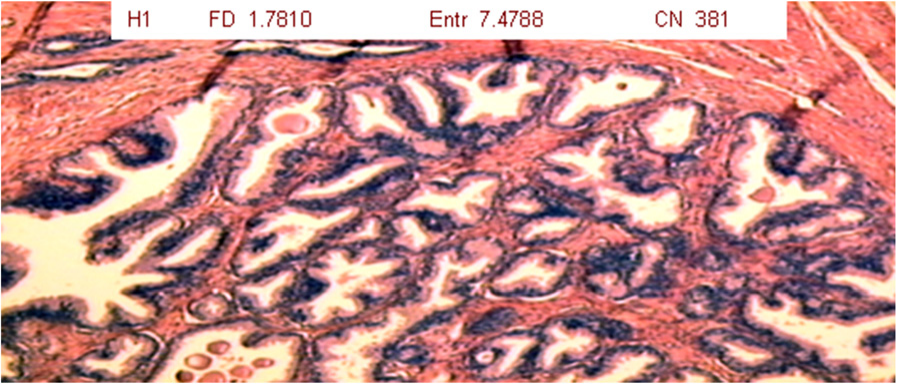
Photomicrograph of hyperplastic prostate tissue showing formation of nodules, denser stroma and wrinkling of acini (magnification: 40x; HE staining).

**Figure 4:**
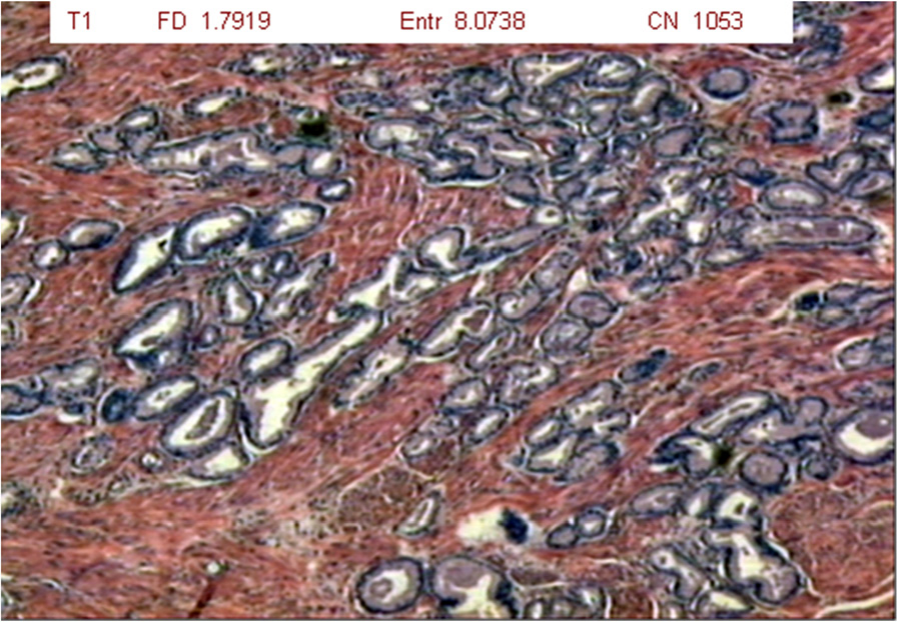
Photomicrograph of tumor prostate tissue showing smaller, clustered acini with infiltration of the muscle tissue (magnification: 40x; HE staining).

The results are expressed considering the fractal dimension, Shannon’s entropy and number of cell nuclei (Table 1). For each patient, slides were analyzed at three different magnifications (1 = 40x, 2 = 100x and 3 = 400x) of tumor tissue (T group – 34 slides), benign hyperplastic tissue (H group – 34 slides) and normal prostate tissue (N group – 34 slides) (Figures 2, 3 and 4).

**Table 1:** Summary of results obtained with significant differences in comparative analyses of fractal dimension, Shannon’s entropy and number of cell nuclei between normal, hyperplastic and tumor tissues

	**N1**	**H1**	**T1**
N1		FD 0.0414	FD 0.0472
	-	SE 0.8691	SE 0.9626
		CN 0.9918	CN 0.0109
H1	FD 0.0414		FD 0.9626
	SE 0.8691	-	SE 0.9626
	CN 0.9918		CN 0.0008
T1	FD 0.0472	FD 0.008	
	SE 0.9626	SE 0.9626	-
	CN 0.0109	CN 0.008	
	**N2**	**H2**	**T2**
N2		FD 0.0005	FD 0.005
	-	SE 0.9473	SE 0.0389
		CN 0.9343	CN 0.001
H2	FD 0.0005		FD 0.5152
	SE 0.9473	-	SE 0.0172
	CN 0.9343		CN 0.001
T2	FD 0.005	FD 0.5152	
	SE 0.0389	SE 0.0172	-
	CN 0.001	CN 0.001	
	**N3**	**H3**	**T3**
N3		FD 0.0036	FD 0.9413
	-	SE 0.9969	SE < 0.001
		CN 0.2276	CN 0.0001
H3	FD 0.0036		FD 0.0936
	SE 0.9969	-	SE < 0.001
	CN 0.2276		CN 0.029
T3	FD 0.9413	FD 0.0936	
	SE < 0.001	SE < 0.001	-
	CN < 0.0001	CN < 0.029	

FD = fractal dimension, SE = Shannon’s entropy, CN = number of cell nuclei.

N = normal tissue, H = hyperplastic tissue, T = tumor tissue.

p < 0.05 – significant difference.

### Fractal dimension

#### 40x magnification

The fractal dimension analysis at a magnification of 40x revealed that the highest median value was found in H1 (1.542), followed by T1 (1.509) and N1 (1.402). The inter-group analysis using the Kruskal-Wallis test revealed significant differences (p = 0.0214). The paired comparison using the Dwass-Steel-Chritchlow-Fligner test revealed significant differences between T1 and N1 (p = 0.0472) as well as between H1 and N1 (p = 0.0414). Using the Bonferroni correction the significance was not maintained (P < α / 3; P <0.05/3; P <0.0167).

#### 100x magnification

The fractal dimension analysis at a magnification of 100x revealed that the highest median value was found in T2 (1.567), followed by H2 (1.564) and N2 (1.458). The inter-group analysis using the Kruskal-Wallis test revealed significant differences (p = 0.0003). The paired comparison using the Dwass-Steel-Chritchlow-Fligner test revealed significant differences between T2 and N2 (p = 0.005) as well as between H2 and N2 (p = 0.0005). These differences were statistically significant after Bonferroni's correction (P < α / 3; P <0.05/3; P <0.0167).

#### 400x magnification

The fractal dimension analysis at a magnification of 400x revealed that the highest median value was found in H3 (1.368), followed by T3 (1.354) and N3 (1.335). The inter-group analysis using the Kruskal-Wallis test revealed significant differences (p = 0.0077). The paired comparison using the Dwass-Steel-Chritchlow-Fligner test revealed a significant difference between H3 and N3 (p = 0.0036). Using the Bonferroni correction the significance was maintained (P < α / 3; P <0.05/3; P <0.0167).

### Entropy

#### 40x magnification

The entropy analysis at a magnification of 40x revealed that the highest median value was found in H1 (7.975), followed by N1 (7.906) and T1 (7.865). The inter-group analysis using the Kruskal-Wallis test revealed no significant differences (p = 0.703).

#### 100x magnification

The entropy analysis at a magnification of 100x revealed that the highest median value was found in T2 (8.168), followed by N2 (8.066) and H2 (8.040). The inter-group analysis using the Kruskal-Wallis test revealed significant differences (p = 0.0107). The paired comparison using the Dwass-Steel-Chritchlow-Fligner test revealed significant differences between T2 and H2 (p = 0.0172) as well as between T2 and N2 (p = 0.0389). Using the Bonferroni correction the significance was not maintained (P < α / 3; P <0.05/3; P <0.0167).

#### 400x magnification

The entropy analysis at a magnification of 400x revealed that the highest median value was found in T3 (8.466), followed by N3 (7.942) and H3 (7.811). The inter-group analysis using the Kruskal-Wallis test revealed significant differences (p = 0.001). The paired comparison using the Dwass-Steel-Chritchlow-Fligner test revealed significant differences between T3 and H3 (p < 0.001) as well as between T3 and N3 (p < 0.001). The statistical significance was maintained in both comparisons after Bonferroni's correction (P < α / 3; P <0.05/3; P <0.0167).

### Cell nuclei

#### 40x magnification

The analysis of cell nuclei at a magnification of 40x revealed that the highest median value was found in T1 (536), followed by H1 (365) and N1 (327). The inter-group analysis using the Kruskal-Wallis test revealed significant differences (p = 0.0008). The paired comparison using the Dwass-Steel-Chritchlow-Fligner test revealed significant differences between T1 and H1 (p = 0.0008) as well as between T1 and N1 (p = 0.0109). Using the Bonferroni correction the significance was maintained in both comparisons (P < α / 3; P <0.05/3; P <0.0167).

#### 100x magnification

The analysis of cell nuclei at a magnification of 100x revealed that the highest median value was found in T2 (571.5), followed by N2 (377.0) and H2 (375). The inter-group analysis using the Kruskal-Wallis test revealed significant differences (p = 0.0001). The paired comparison using the Dwass-Steel-Chritchlow-Fligner test revealed significant differences between T2 and H2 (p < 0.0001) as well as between T2 and N2 (p < 0.0001). The statistical significance was maintained in both comparisons after Bonferroni's correction (P < α / 3; P <0.05/3; P <0.0167).

#### 400x magnification

The analysis of cell nuclei at a magnification of 400x revealed that the highest median value was found in T3 (168.5), followed by H3 (126) and N3 (108). The inter-group analysis using the Kruskal-Wallis test revealed significant differences (p = 0.0001). The paired comparison using the Dwass-Steel-Chritchlow-Fligner test revealed significant differences between T3 and H3 (p = 0.0029) as well as between T3 and N3 (p < 0.0001). Using the Bonferroni correction the significance was maintained in both comparisons (P < α / 3; P <0.05/3; P <0.0167).

## Discussion

The results of the fractal dimension, entropy and number of cell nuclei demonstrate that such analyses can contribute toward the diagnosis of prostate cancer. The fractal dimension analysis of the histological slides revealed that the highest median values at magnifications of 40x and 400x were obtained in the prostate tissue with benign hyperplasia, whereas the lowest values were obtained in normal tissue. Significant differences were found between the T and N groups at 40x and 100x as well as between the H and N groups at 40x at 400x. After utilizing the Bonferroni correction, the differences were significant at magnifications of 100x and 400x. Among the three magnifications studied, 100x differed in the T group, suggesting that this degree of magnification may be used in clinical practice. However, this magnification did not constitute satisfactory parameter for differentiating hyperplastic tumors.

These findings are in agreement with data described by Tambasco *et al*., [7] who investigated the architectonic complexity of fragments from 63 patients with benign prostate tissue and 19 patients with high-grade carcinoma based on histological slides and found that the mean of the fractal dimension was higher in the group with high-grade carcinoma. In the present study, the three types of tissue analyzed (normal, benign hyperplastic and tumor) were from the same patient and same gland. Moreover, the tumors studied herein were selected randomly, with no knowledge on the histological grade.

Tambasco *et al*. [7] report 84.2 and 89.5% sensitivity and 82.5% and 90.5% specificity using hematoxylin-eosin (HE) and pan-keratin, respectively, in the comparison of benign prostate tissue and high-grade carcinoma. These differences may be due to the fact that pan-keratin is more specific for glandular tissue than HE, which was the technique used in the present study. Moreover, the authors cited only used high-grade carcinoma (Gleason 8 to 10). Investigating the detection of prostate cancer using ultrasound on seven patients, Moradi *et al*. [9] found a low degree of specificity (61.9%), suggesting that this diagnostic imaging method is not as specific as the results obtained with the processing of images of histological slides.

The present results suggest the possibility of using fractal dimension analysis in the diagnosis of prostate cancer, as exams such as the determination of PSA exhibit a low degree of specificity (25 and 33%), [10,11] generating doubts regarding the actual need for a biopsy. According to Arruda & Arruda, [12] there are divergent opinions regarding the importance of the PSA exam in the diagnosis of prostate cancer, as this antigen does not offer all the characteristics of an ideal tumor marker.

Investigating the computerized detection of prostate cancer on T2-weighted magnetic resonance images with a combination between fractal and multifractal features to perform textural analysis of the image, Lopes *et al*. [13] found that method was more accurate than the classical texture based method.

Quantifying the complexity of the epithelial-conjunctive tissue interface of 377 normal, dysplastic and neoplastic human oral mucosae using digital images and applying the box-counting method to estimate the fractal dimension, Abu Eid & Landini [14] found significant differences been normal, pre-malignant and malignant tissues. The authors concluded that fractal geometry is useful in the evaluation alterations of the tissue complexity that occur due to malignant transformations and can be used as a quantitative marker of epithelial complexity.

Fractal geometry can also provide data to forge a consensus among pathologists on a relatively large amount of cases of diagnostic doubt, thereby minimizing variability regarding medical opinion. Moreover, this method can serve as a screening tool, identifying low-grade tumors and reducing the time pathologists spend on the study of areas of compromised tissue [7].

The results of the entropy analysis revealed the highest median value was obtained in the hyperplastic tissue at a magnification of 40x and in the tumor tissue at 100x and 400x. In the comparison between groups, significant differences were found between the T x N groups at 100x and 400x as well as between the T and H groups at 100x and 400x. However, after utilizing the Bonferroni correction the difference was only significant at 400x Thus, magnification at 400x differentiated tumor tissue from both normal tissue and hyperplastic tissue, indicating the possibility of using these degrees of magnification in the identification of prostate cancer.

As Shannon’s entropy quantifies the degree of complexity in information (as that contained in histological slides), there is a high probability of differentiating tumor tissue from normal tissue, indicating that this method could be useful in the diagnosis of prostate cancer. Yogesan *et al*. [15] carried out the only study in the literature on the diagnostic contribution of the calculation of entropy in nuclear images of cases of prostate cancer, demonstrating the possibility of using entropy analysis for differentiating cases of a good prognosis from those with a poor prognosis. Investigating the applicability of the calculation of Shannon’s entropy in the evaluation of the texture of images in normal and abnormal regions of digital mammograms, Pharwaha & Singh [16] concluded that this method is useful in the diagnosis of breast cancer.

The results of the analysis of cell nuclei revealed that the highest median values at all three magnifications occurred in tumor tissue. The paired comparison revealed significant differences between the T and N groups as well as between the T and H groups at all three magnifications. Thus, the three degrees of magnification differentiated tumor tissue from both normal and hyperplastic tissue, achieving a better performance than the fractal dimension and entropy analyses. The findings support the use of the analysis of cell nuclei at these different degrees of magnification in the diagnosis of prostate cancer. As the number of cell nuclei is higher with a greater degree of differentiation due to the physiopathologic mechanisms of tumor growth and tissue infiltration, this method can be used in the histological diagnosis and decisions regarding the best form of treatment.

No studies were found in the literature on the use of Shannon’s entropy and the analysis of cell nuclei in the diagnosis of prostate cancer. Considering the fact that this form of cancer has the second highest incidence among men, [17] fractal dimension analysis can contribute toward clarifying the histological diagnosis of prostate cancer based on biopsies used in the detection of this disease and the determination of the Gleason classification, as such information is often dubious and depends on the subjective opinion of the pathologist.

## Conclusions

The quantification of the fractal dimension, Shannon’s entropy and cell nuclei can contribute toward the histopathological diagnosis of prostate cancer. In the fractal dimension analysis, magnification at 40x and 100x differentiated tumor tissue from normal prostate tissue. In the calculation of Shannon’s entropy, magnification at 100x and 400x differentiated tumor tissue from both normal and hyperplastic tissue. The quantification of cell nuclei allowed the differentiation of tumor tissue from both normal and hyperplastic tissue at all three degrees of magnification studied, indicating potential in clinical practice for the histological diagnosis of prostate cancer.

## Competing interests

The authors declare having no competing interests.

## Authors’ contributions

PFFA contributed to the conception and design, the acquisition, analysis and interpretation of the data, drafting of the final manuscript, and final approval of the version to be published. MG contributed to the acquisition, analysis and interpretation of the data. FNFJ contributed to the drafting of the final manuscript. JGFA contributed to the final approval of the version to be published. RDM contributed to the acquisition, analysis and interpretation of the data. LOM contributed to the improvement of the image software and drafting of the final manuscript. LFA contributed to the analysis and interpretation of the data and drafting of the final manuscript. MFG contributed to the conception and design, the analysis and interpretation of the data, drafting of the final manuscript, and final approval of the version to be published. All authors contributed to the drafting of the final manuscript, which they read and approved.

## Pre-publication history

The pre-publication history for this paper can be accessed here:

http://www.biomedcentral.com/1472-6890/13/6/prepub
